# Preparation of Polyvinylidene Fluoride (PVDF) Hollow Fiber Hemodialysis Membranes

**DOI:** 10.3390/membranes4010081

**Published:** 2014-02-27

**Authors:** Qinglei Zhang, Xiaolong Lu, Lihua Zhao

**Affiliations:** 1 Institute of Biological and Chemical Engineering, Tianjin Polytechnic University, Tianjin 300387, China; E-Mails: haiyang19802005@163.com (Q.Z.); 13516211811@139.com (L.Z.); 2 State Key Laboratory of Hollow Fiber Membrane Materials and Membrane Processes, Tianjin Polytechnic University, Tianjin 300387, China

**Keywords:** PVDF, non-solvent-induced phase separation, hollow fiber hemodialysis membrane, polyethylene glycol, performance of membrane

## Abstract

In this study, the polyvinylidene fluoride (PVDF) hollow fiber hemodialysis membranes were prepared by non-solvent induced phase separation (NIPS). The influences of PVDF membrane thickness and polyethylene glycol (PEG) content on membrane morphologies, pore size, mechanical and permeable performance were investigated. It was found that membrane thickness and PEG content affected both the structure and performance of hollow fiber membranes. The tensile strength and rejection of bovine serum albumin (BSA) increased with increasing membrane thickness, while the Ultrafiltration flux (UF) flux of pure water was the opposite. The tensile strength, porosity and rejection of BSA increased with increasing PEG content within a certain range. Compared with commercial F60S membrane, the PVDF hollow fiber membrane showed higher mechanical and permeable performance. It was proven that PVDF material had better hydrophilicity and lower BSA adsorption, which was more suitable for hemodialysis. All the results indicate that PVDF hollow fiber membrane is promising as a hemodialysis membrane.

## 1. Introduction

Hemodialysis (HD) is a relatively safe purification technique for curing renal failure. Excess moisture and metabolic wastes (such as urea and creatinine) were removed by HD, and in the meantime, calcium ion, bicarbonate ion and other substances can be supplied. The core element is ultrafiltration hollow fiber membrane (HFM) [1]. Currently, polyethersulfone (PES) and polysulfone (PSf) membranes are widely used in hemodialysis for their better biocompatibility and functional middle-molecular substance clearances [2]. However, the biocompatibility of these membranes is still not ideal and needs improvement [3,4]. For example, anticoagulants (such as hirudin or heparin) should be added during hemodialysis, owing to the poor anticoagulation property of commercial membranes [5]. Studies on developing high performance hemodialysis membranes have attracted worldwide attention. So far, many works have been focused on the modification of current membranes for the purpose of enhancing their hemodialysis properties. The most widely used method for improving biocompatibility is to use additives that have excellent biocompatibility rather than the native polymer. PSf blended with polyvinylpyrrolidone (PVP) showed enhanced biocompatibility compared to native PSf [6]. Although the modification of the currently used materials is an effective way to improve the biocompatibility of hemodialysis membranes, it is far from being clinically applicable, owing to the complexity of the modification process. Therefore, it is urgently needed to find new materials with promising biocompatibility and hemodialysis properties.

Polyvinylidene fluoride (PVDF), a widely used material in the field of water purification, has recently received great attention as a membrane material with regard to its outstanding properties, such as high mechanical strength, thermal stability, anti-ultraviolet radiation, smooth surface and low protein adsorption [7,8,9,10,11,12]. Laroche *et al*. [13] pointed out that PVDF had excellent biocompatibility and minimal cell adsorption and tissue response. PVDF has a promising future application in the hemodialysis field. However, the intrinsic hydrophobicity of PVDF limits the practical application in biomedicine [6,14]. Therefore, it is essential to improve the hydrophilicity of the material surface.

Polyethylene glycol (PEG) containing copolymers has been extensively investigated to modify the surface property of various industrial membranes, because PEG is considered to be one of the best synthetic non-fouling materials that has the ability to resist protein adsorption. The anti-fouling performance of PEG can be attributed to its unique properties of having a high level of hydrophilicity, vigorous chain mobility and a high extent of coordination with surrounding water molecules [15].

An attempt was made to improve the mechanical and permeable properties of PVDF hollow fiber membranes by blending with PEG polymers in this study. At the same time, the preliminary biocompatibility evaluation of materials was studied. Additionally, the specific biocompatibility evaluation of PVDF hollow fiber membranes will be expanded in the next work. The effects of PEG content on membrane morphology were evaluated by scanning electron microscopy (SEM). Furthermore, the influences of different membrane thickness and PEG content on membrane properties were also investigated. The permeation and hydrophilicity of the membrane surface were evaluated by the UF flux of pure water and the water contact angle. The protein rejection and protein adsorption were investigated using bovine serum albumin (BSA).

## 2. Materials and Methods

### 2.1. Materials

Polyvinylidene fluoride (PVDF 1010, SOLVAY, Lyon, France), polyethylene glycol (Tianjin Fukang Chemical Company, Tianjin, China), *N*,*N*-dimethylacetamide (Samsung Company, Seoul, Korea) and bovine serum albumin (Shanghai Biomedical Engineering Technical Service Company, Shanghai, China) were used. All the reagents used in the study were of reagent grade.

### 2.2. Preparation of PVDF Hollow Fiber Membranes

**Figure 1:**
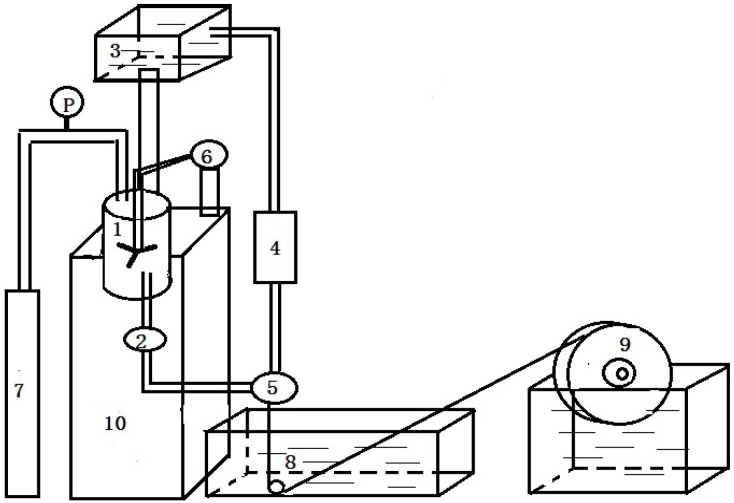
The schematic of polyvinylidene fluoride (PVDF) hollow fiber spinning equipment. 1: dope tank; 2: metal filter; 3: liquid tank; 4: flow meter; 5: spinneret; 6: agitator; 7: nitrogen cylinder; 8: coagulation bath; 9: take-up wheel; 10: control device.

The PVDF hollow fiber membranes were prepared by non-solvent-induced phase separation (NIPS) through spinning equipment, as shown in Figure 1. Table 1 lists the spinning parameters. Casting dopes were prepared by adding PEG (6000 Da) into the solvent, including PVDF, 1,4-diethylene dioxide and *N*,*N*-dimethylacetamide (DMAc), followed by stirring at 70 °C until the solution became homogeneous. 1,4-diethylene dioxide and PEG worked as pore-forming agents to produce porous structures in the membrane. The dope solution was then transferred into a tank and kept at a constant temperature of 70 °C for 12 h to eliminate the air bubbles in the solution before being used. The casting solution and bore fluid passed through the orifice and inner tube, respectively. The nascent membranes were taken up at a drawing rate of 80 m·min^−1^ and immersed in UF water for at least 48 h to remove the residual DMAc, then kept in glycerol aqueous solution with a specific gravity of 1.08 for 48 h to prevent the collapse of porous structures. Finally, the membranes were dried in ambient air until ready to use. The inner diameter and different wall thicknesses of PVDF hollow fiber membranes are 200 and 30, 40 and 50 μm, respectively.

**Table 1:** The preparation conditions for solution-cast membranes. PEG = Polyethylene glycol.

Membrane label	PVDF (wt %)	PEG (6000 Da) (wt %)	1,4-diethylene dioxide (wt %)	DMAc (wt %)	Viscosity mPa·s
F24-a	24	14.8	25.2	36	2936
F24-b	24	16.8	25.2	34	4124
F24-c	24	18.8	25.2	32	4376

### 2.3. Characterization of the Hollow Fiber Membranes

#### 2.3.1. Morphology of Membranes and Measurement of Pore Size and Porosity

Morphology studies of PVDF HFMs were carried out using a scanning electron microscope (Hitachi S-4800, HITACHI, Tokyo, Japan). The wet membranes were immersed in liquid nitrogen and fractured carefully. The specimen were put on a metal support and dried under vacuum for 24 h. Then, the specimen was coated by sputtering gold under vacuum using a Bal-Tec SCD 005 sputter coater (HITACHI, Tokyo, Japan). Samples were observed under an electron microscope at 10 kV.

**Figure 2:**
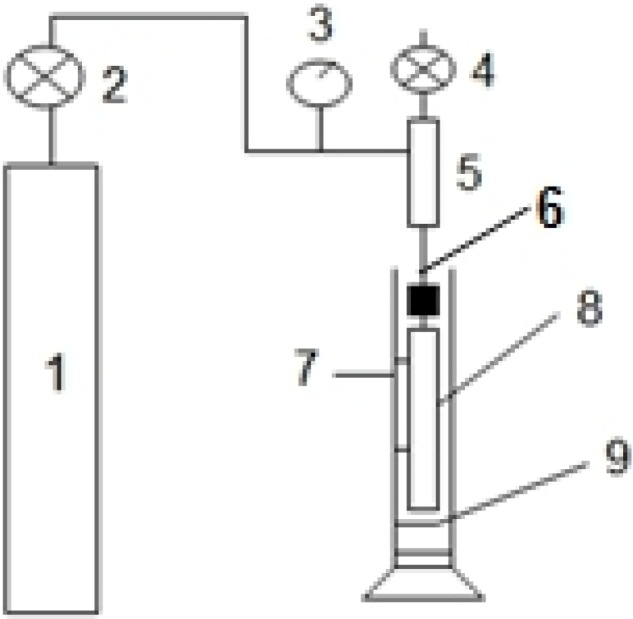
The apparatus for determining the maximum pore size of the hollow fiber membranes. 1: nitrogen bottle; 2: regulator; 3: precise pressure gauge; 4: valve; 5: container; 6: syringe needles; 7: Transparent cylinder; 8: PVDF membrane sample to be tested; 9: absolute ethyl alcohol.

The maximum pore size was determined using equipment, as shown in Figure 2. Measurements were carried out on fiber soaked in ethanol for 15 min. At room temperature, the membrane was immersed in ethanol, and then, nitrogen can be pressurized into the inside of the membranes. The bubble point pressure, *P*, is reached when the first string of bubbles comes from the walls of the membranes. The maximum pore size can be calculated according to the following Equation (1) [16]:

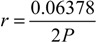
(1)
where *r* is the pore radius (μm), *P* is bubble point pressure (MPa), and the ethanol surface tension is 22.3 mN/m.

The membrane porosity, ε, was measured by soaking the membrane in pure water for 2 h, and then, the membrane surface was dried by filter paper. The membrane was weighed before and after absorption of the pure water. The porosity was calculated using Equation (2):

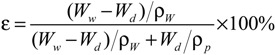
(2)
where ε is the porosity of the membrane (%), *W_w_* is the mass of the wet membrane, *W_d_* is the mass of the dry membrane, ρ*_W_* is the density of water (1.0 g/cm^3^) and ρ*_p_* is the density of the membrane (1.78 g/cm^3^, as reported in Solvay technical sheets [17]).

#### 2.3.2. Mechanical Properties

The mechanical properties of the fabricated membranes were measured using an electronic single yarn strength tester (YG061 F/PC, Lanzhou Electron Instrument Co., Ltd., Lanzhou, China) at room temperature. Each sample was clamped at both ends and stretched unidirectionally at a constant elongation rate of 500 mm/min with an initial length of 10 cm. Specimens were selected randomly and tested from each batch of the dried hollow fiber sample. The tensile elongation and tensile strength at break were determined.

Bursting pressure [16] is a mechanical performance parameter, which has caused the wall of the structure to change from an initial shape to a damaged shape. The membrane will be damaged with increasing pressure after the bubble point (2.3.1. maximum pore size).

#### 2.3.3. The Pure Water Flux and Rejection of BSA

**Figure 3:**
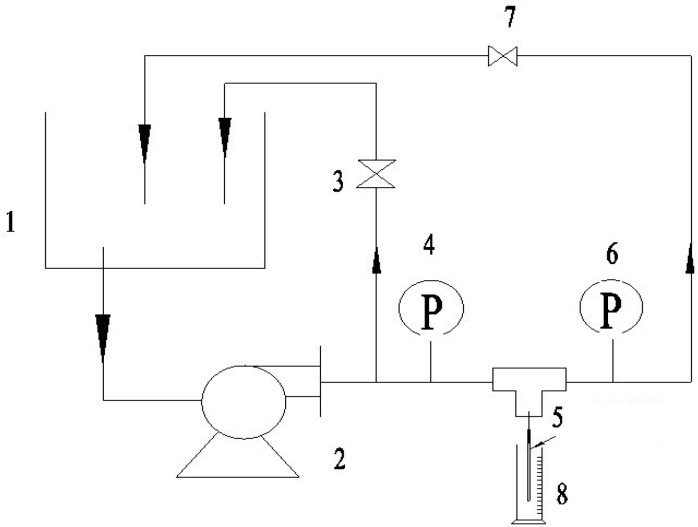
The schematic of the experimental systems used for the UF water flux and rejection of bovine serum albumin (BSA). 1: container; 2: magnetic drive pump; 3,7: valve; 4,6: precision gauge; 5: syringe needle; 8: measuring cylinder.

After adjusting the test temperature (25 °C), the pure water flux at certain transmembrane pressures was measured (as shown in Figure 3) under steady state conditions using the Equation (3):

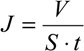
(3)
where *J* is pure water flux (L·h^−^^1^·m^−2^), *V* is the volume of the permeate (L), *S* is effective membrane area (m^2^) and *t* is sampling time (h).

**Figure 4:**
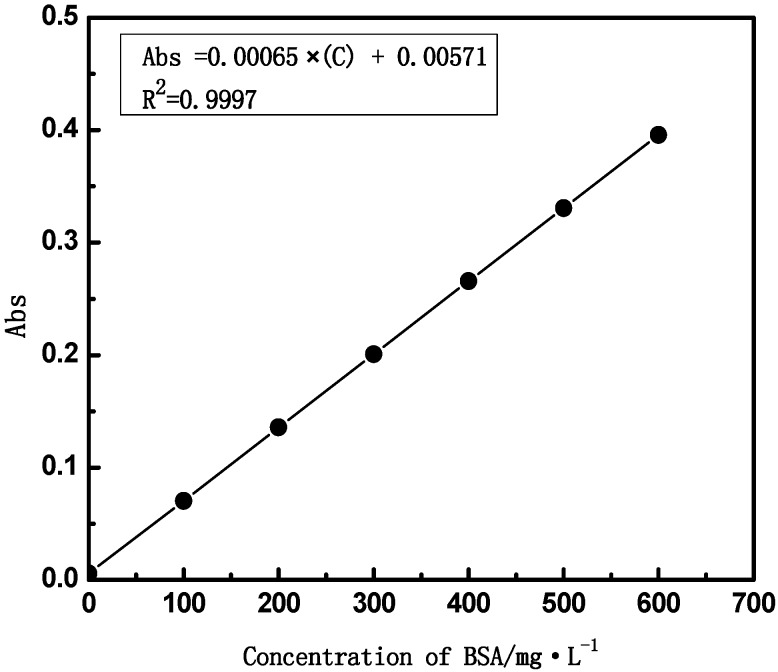
The standard curve of BSA. The concentrations of BSA are measured with a UV-Vis spectrophotometer (TU-1810, Purkinje, Beijing, China).

The rejection ratio of BSA(*R*) was calculated by the following Equation (4):

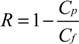
(4)
where *C_p_* and *C_f_* (mg·L^−1^) are BSA concentrations of the permeate and the remaining solutions, respectively. The concentration of BSA is determined by the standard curve of BSA, as shown in Figure 4.

### 2.4. Biocompatibility of PVDF Hollow Fiber Membrane

#### 2.4.1. Water Contact Angle

The water contact angle of the membrane surface was measured by using the sessile drop method, and all the contact angle data were an average of five measurements on different locations of the membrane surface. A water droplet was introduced on the surface of the membranes, and the contour of the water drop was recorded. In this method, by optical microscopy (YH-168A, Harke, Beijing, China), we obtain the profile of a drop deposited on a horizontal surface. The image profile of the drop obtained by the digital camera is analyzed by software (Surftens, Solvent Innovation GmbH, Cologne, Germany), adjusting the diameter of the drop and the contact angle with the surface.

#### 2.4.2. Protein Adsorption

The protein adsorption experiments were carried out with BSA, which were dissolved in the phosphate-buffered saline solution (PBS, pH 7.4) with a concentration of 1 mg·mL^−^^1^, respectively. The membranes with an area of 10 cm × 10 cm were incubated in PBS solution for 24 h and then immersed in the protein solution for 12 h at 25 °C. The BSA concentration before and after membrane adsorption was measured at the wavelength of 278.00 nm. Then, the adsorbed BSA amounts by the membranes were calculated.

## 3. Results and Discussion

### 3.1. Effect of Thickness on Membrane Characterization

#### 3.1.1. Effect of Thickness on Membrane Morphology and Structure

**Figure 5:**
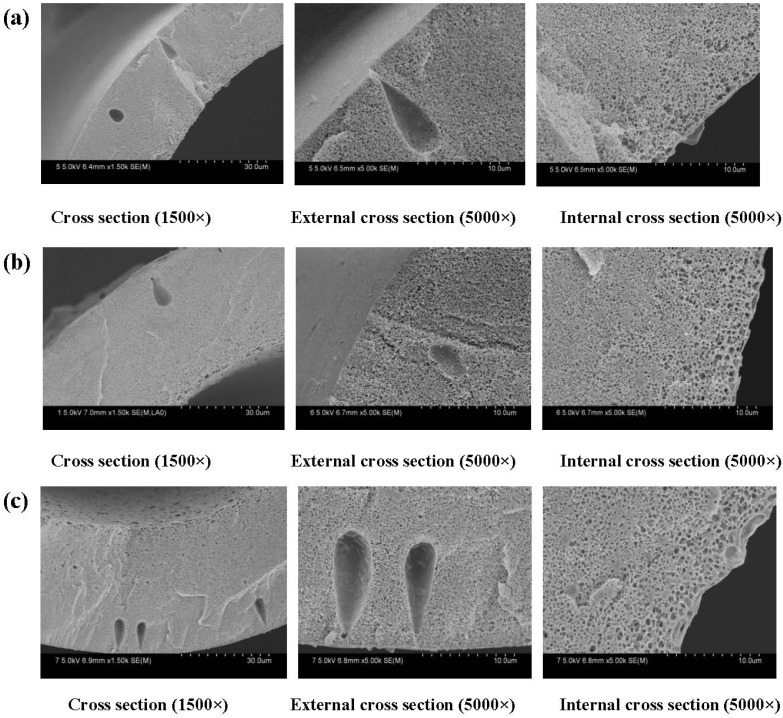
SEM images of different membrane thickness. The inner diameter is 200 μm. The wall thickness of the PVDF hollow fiber membranes: (**a**) 30 μm; (**b**) 40 μm; (**c**) 50 μm. The preparation parameters of PVDF membranes were shown in Table 1 (F24-b).

The cross-section SEM morphologies of PVDF membranes are shown in Figure 5. A series of PVDF membranes were prepared by the phase inversion method. PVDF was the membrane matrix, and PEG was a modifier to enhance the hydrophilicity. The results exhibited that membranes have a typical asymmetric structure with a dense skin layer on outside, an intermediate layer with a finger-like structure and a bottom layer of fully developed pores. This structure is a typical structure that is formed via NIPS, especially by instantaneous phase separation. In the formation of hollow fiber membranes, the polymer solution extruded from the spinneret was immersed in a pure water bath. These experimental membranes have an inner diameter and wall thickness of 200 μm and 30–50 μm, respectively, which are approximately the same size as commercial hemodialysis membranes (200/40 μm).

**Figure 6:**
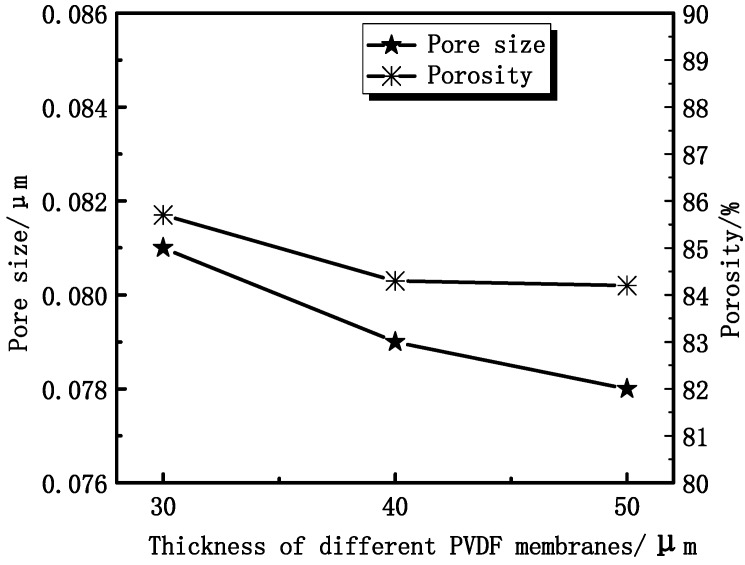
The effect of membrane thickness on the maximum pore size and porosity.

From Figure 6, it can be seen that the maximum diameter decreases from 0.081 μm to 0.078 μm, which is obtained from Equation (6). Experimental results show that the porosity ranges from 86% to 84%, as shown in Figure 6. The porosity of membranes has no significant change, while the maximum diameter of the membrane pore decreases with increasing thickness. That can be explained by the accelerating of the instantaneous phase. The solvent and non-solvent exchange rate increases with the membrane thickness increasing. The outer surface of the membrane has a lager contact area with the non-solvent.

#### 3.1.2. Effect of Thickness on Membrane Mechanical Properties

**Figure 7:**
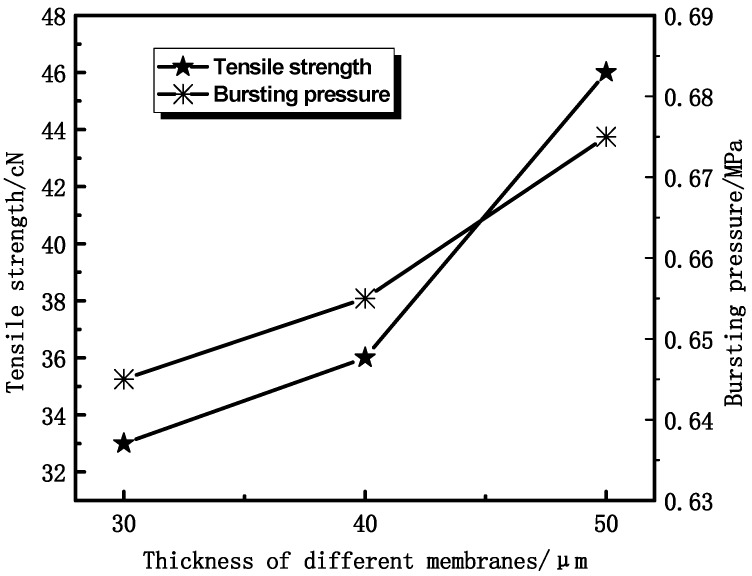
The effect of thickness on tensile strength and bursting pressure.

**Figure 8:**
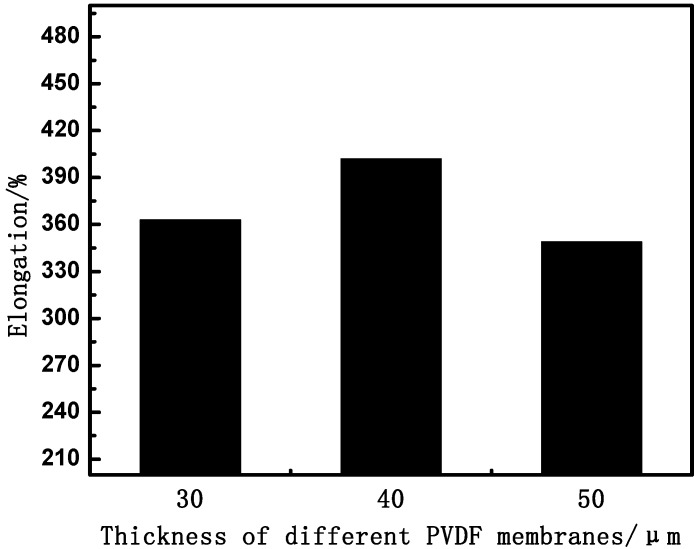
The effect of thickness on tensile elongation.

In view of the potential practical applications in the biomedical field, it is essential for PVDF HFMs to retain good mechanical strength. To evaluate the mechanical properties of the PVDF membranes, the tensile properties of all samples are exhibited in Figure 7 and Figure 8. It can be seen that the tensile strength and bursting pressure increase with the thickness increasing. The tensile elongation of the membranes was little changed with thickness increasing, as shown in Figure 8, which is mainly due to the similar structure of the materials.

#### 3.1.3. Effect of Thickness on Membrane Permeation Performance

**Figure 9:**
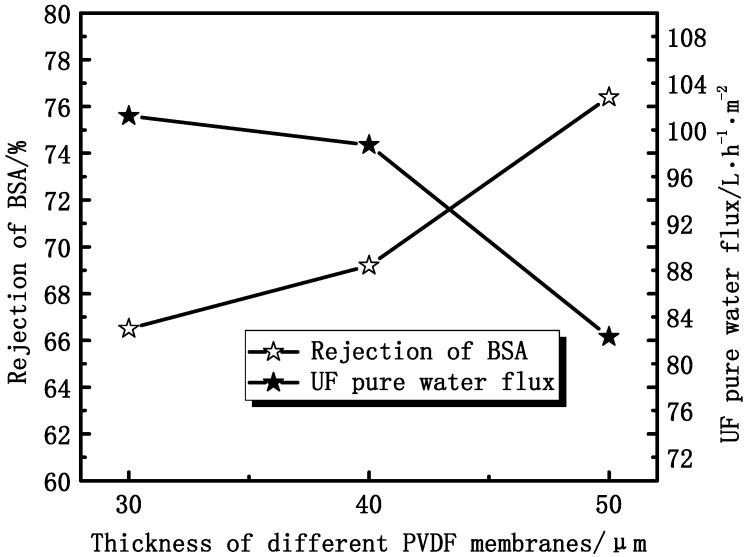
The effect of thickness on the UF flux and the rejection of BSA.

As shown in Figure 9, the UF flux decreases from 101.2 L·h^−1^·m^−2^ to 82.3 L·h^−1^·m^−2^, while the rejection ratio of BSA increases from 66.5% to 76.4%, with the membrane thickness increasing. The UF water flux reduction is mainly due to the increase of the transmembrane resistance with the membrane thickness increasing. The increase of the rejection ratio can be due to the different operating environments.

### 3.2. Effect of PEG Content on Membranes Performance

#### 3.2.1. Effect of PEG Content on Membranes Morphology and Structure

**Figure 10:**
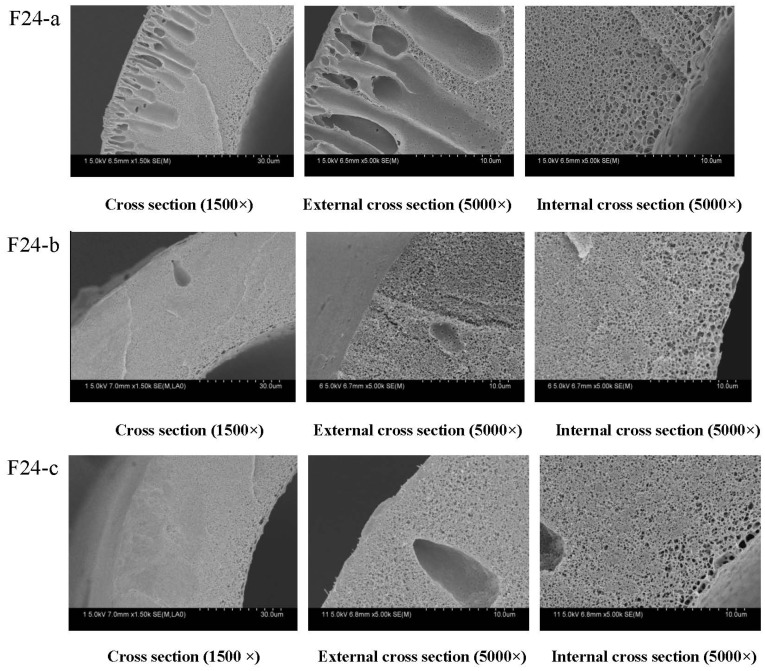
The effect of PEG content on the microstructure of PVDF hollow fiber membrane percentage: (**a**) 14.8% (w/w); (**b**) 16.8% (w/w); (**c**) 18.8% (w/w). The preparation parameters of PVDF membranes were shown in Table 1. The inner diameter and different wall thickness of PVDF hollow fiber membranes are 200 and 40 μm, respectively.

Figure 10 shows the SEM results of cross-sectional structures of PVDF hollow fiber membranes. From Figure 10, it can be seen that the cross-sectional structures of the membranes are different. Long finger-like pores are present near the outer walls of the hollow fiber membranes, while sponge-like structures are possessed by the center of the hollow fiber membranes and the inner walls. The appearance of the fiber structure can be due to the rapid precipitation resulting in finger-like pores and the slow precipitation giving the sponge-like structure. When the PEG content was 14.8% (mass fraction), finger-like pores dominated the cross-section. When the PEG content was 16.8% and 18.8%, the finger-like structure was changed to a sponge-structure. The casting solution viscosity can affect the solvent and non-solvent diffusion rate, which delays the occurrence of phase separation and inhibits the generation of the finger holes. As the PEG concentration was increased from 14.8% to 18.8%, the viscosity of the doping solution changed dramatically. The finger-like structure was changed to the sponge-structure formed by the delayed phase separation.

**Figure 11:**
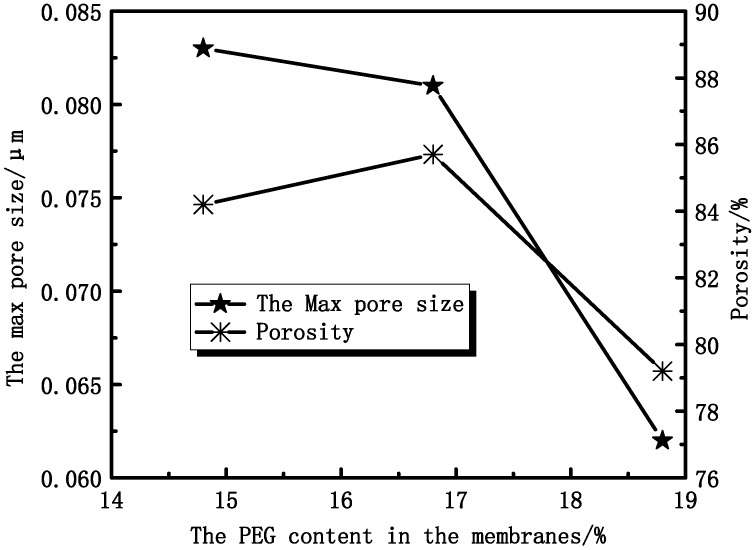
The effect of PEG content on membrane maximum pore size and porosity.

From Figure 11, it can be found that the maximum diameter of the membrane pore ranges from 0.083 μm to 0.062 μm. Experimental results show that the porosity of membranes increases at first and then decreases. That can be explained by considering the casting solution viscosity, which can affect the solvent and non-solvent diffusion rate.

#### 3.2.2. Effect of PEG Content on Membrane Mechanical Properties

**Figure 12:**
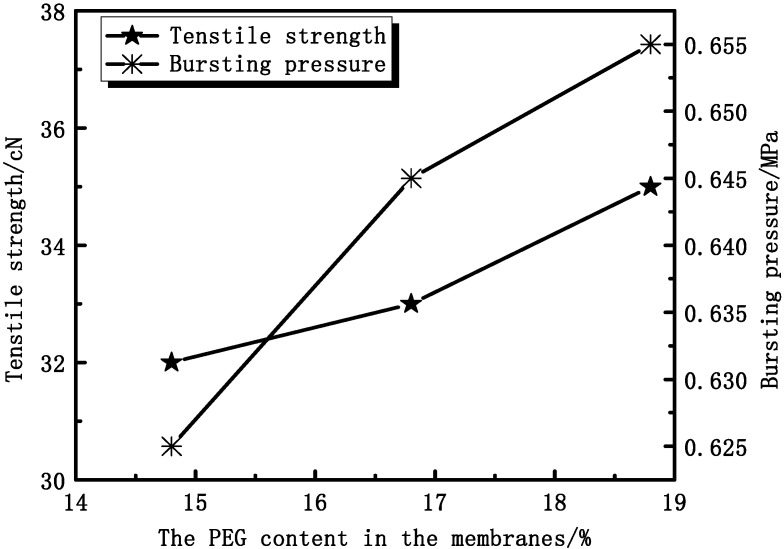
The effect of PEG content on tensile strength and bursting pressure.

As Figure 12 shows, the tensile strength and bursting pressure increase with increasing PEG content. The tensile strength increases from 32 cN to 35 cN and the bursting pressure increases from 0.625 MPa to 0.655 MPa. As can be seen from SEM (Figure 10), the proportion of the sponge-like structure increases. With increasing PEG content, the casting solution viscosity is increased. As a result, the molecular movement is restricted, which is difficult for forming more pores in membranes.

#### 3.2.3. Effect of PEG Content on Membranes Permeation Performance

**Figure 13:**
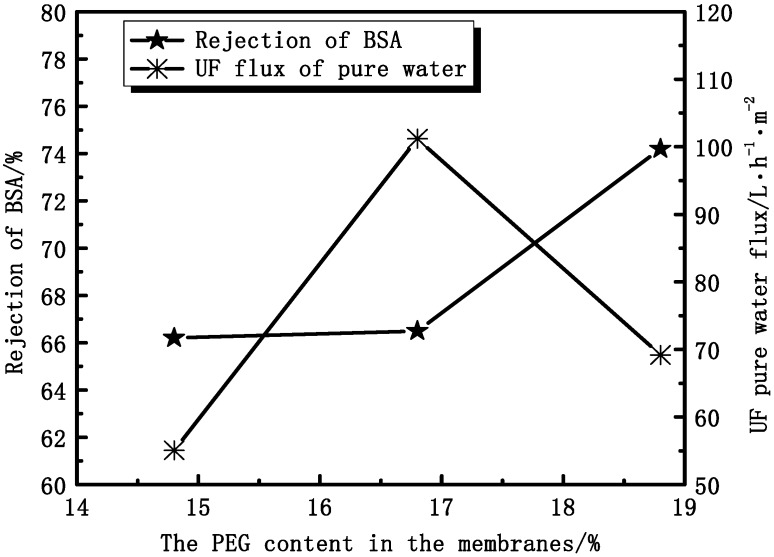
The effect of PEG content on the UF flux and the rejection of BSA.

In the same conditions, different contents of PEG in the membranes affect the UF flux and the rejection of BSA. Figure 13 shows that the rejection of BSA increases with increasing PEG content, while the UF flux increases at first and then decreases. At the point of 16.8%, the UF flux of pure water has the max value. The UF flux mainly depends on the effective pores instead of the porosity. The rejection ratio of BSA depends more on the denseness of the skin layer than the structure of the cross-section. Thus, the skin layers of PVDF/PEG blend membranes are denser and denser with increasing thickness.

### 3.3. Comparison with Commercial F60S Hemodialysis Membrane

#### 3.3.1. Mechanical and Permeation Properties

**Figure 14:**
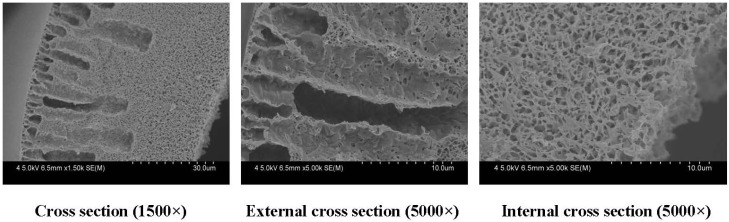
The cross-section of F60S membrane by SEM; the inner diameter and different wall thickness of PVDF hollow fiber membranes are 200 and 40 μm, respectively.

Figure 14 shows the SEM results of cross-sectional structures of F60S hollow fiber membrane. There are finger-like pores, mainly at the outer layer of the membrane. The sponge-like structure can be detected at the inner layer of the fiber. The SEM cross-section of the PVDF hollow fiber membrane shows few finger-like structures (F24-b).

From the Table 2, it can be seen that the PVDF hollow fiber membrane has better mechanical performance, higher porosity and pure water flux than the F60S membrane, while the rejection of BSA is lower. The different materials have different mechanical properties, which mainly depend on the different cross-section of the membranes.

**Table 2:** The mechanical and permeation performance of the PVDF and F60S membranes.

Materials	Tensile strength cN	Elongation %	Bursting pressure MPa	Pore size μm	Porosity %	UF flux L/(h·m^2^)	Rejection of BSA %
PVDF	40	402	0.645	0.079	84.3	98.7	69.2
F60S	27	66	0.475	0.079	72.3	72.5	78.2

#### 3.3.2. Water Contact Angle and BSA Adsorption

The water contact angle is a convenient way to assess the wettability properties of the membrane surface. When the membrane is used for blood separation, protein adsorption is the first stage of the interactions of membrane and blood, which may lead to undesirable results. Therefore, it is an important factor to evaluate the blood compatibility of a material. Table 3 shows that the PVDF membrane and the F60S membrane were both hydrophilic materials, but the water contact angle of the PVDF membrane is smaller than F60S. The PVDF membrane has better hydrophilicity and less BSA adsorption than the F60S membrane. This is because the F-C bond energy of PVDF is high, and the fluorine atom size is small. At the same time, polyethylene glycol (PEG) is an uncharged water-soluble polymer with hydrophilicity and a large exclusion volume, and it has the extraordinary ability to resist protein adsorption.

**Table 3:** Water contact angle and BSA adsorption of the PVDF and F60S membranes.

Materials	Water contact angle (°)	BSA adsorption (mg/m^2^)
PVDF	54 ± 3	145 ± 3
F60S	64 ± 2	235 ± 2

## 4. Conclusions

The membrane thickness and PEG content can affect the mechanical and permeation properties of PVDF hollow fiber membranes. The mechanical properties and rejection of BSA increase while UF pure water flux decreases with increasing membrane thickness. The mechanical properties and rejection of BSA increase with increasing PEG content.

In this work, PVDF hollow fiber membranes were produced by NIPS, using PEG (6000 Da) as a pore-forming additive. Through the optimization experiments, the membrane thickness (40 μm) was determined, and the PEG content was 16.8%. Compared with commercial F60S membranes, the PVDF hollow fiber membrane has greater advantages in the mechanical properties and ultra-filtration water flux. The PVDF membrane shows better hydrophilicity and lower BSA adsorption. All the results indicate that PVDF hollow fiber membrane is promising as a hemodialysis membrane. 
